# Three-dimensional imaging for the quantification of spatial patterns in microbiota of the intestinal mucosa

**DOI:** 10.1073/pnas.2118483119

**Published:** 2022-04-27

**Authors:** Octavio Mondragón-Palomino, Roberta Poceviciute, Antti Lignell, Jessica A. Griffiths, Heli Takko, Rustem F. Ismagilov

**Affiliations:** ^a^Division of Chemistry and Chemical Engineering, California Institute of Technology, Pasadena, CA 91125;; ^b^Division of Biology and Biological Engineering, California Institute of Technology, Pasadena, CA 91125

**Keywords:** microbiota, quantitative biogeography, tissue clearing

## Abstract

Improving our understanding of host–microbe relationships in the gut requires the ability to both visualize and quantify the spatial organization of microbial communities in their native orientation with the host tissue. We developed a systematic procedure to quantify the three-dimensional (3D) spatial structure of the native mucosal microbiota in any part of the intestines with taxonomic and high spatial resolution. We performed a 3D biogeographical analysis of the microbiota of mouse cecal crypts at different stages of antibiotic exposure. By tracking eubacteria and four dominant bacterial taxa, we found that the colonization of crypts by native bacteria is a dynamic and spatially organized process. Ciprofloxacin treatment drastically reduced bacterial loads and eliminated Muribaculaceae (or all Bacteroidetes entirely) even 10 d after recovery when overall bacterial loads returned to preantibiotic levels. Our 3D quantitative imaging approach revealed that the bacterial colonization of crypts is organized in a spatial pattern that consists of clusters of adjacent colonized crypts that are surrounded by unoccupied crypts, and that this spatial pattern is resistant to the elimination of Muribaculaceae or of all Bacteroidetes by ciprofloxacin. Our approach also revealed that the composition of cecal crypt communities is diverse and that Lactobacilli were found closer to the lumen than Bacteroidetes, Ruminococcaceae, and Lachnospiraceae, regardless of antibiotic exposure. Finally, we found that crypts communities with similar taxonomic composition were physically closer to each other than communities that were taxonomically different.

## Significance

Many human diseases are causally linked to the gut microbiota, yet the field still lacks mechanistic understanding of the underlying complex interactions, because existing tools cannot simultaneously quantify microbial communities and their native context. In this work, we provide an approach to tissue clearing and preservation that enables 3D visualization of the biogeography of the host–microbiota interface. We combine this tool with sequencing and multiplexed microbial labeling to provide the field with a platform on which to discover patterns in the spatial distribution of microbes. We validated this platform by quantifying bacterial distribution in cecal mucosa at different stages of antibiotic exposure. This approach may enable researchers to formulate and test new hypotheses about host–microbe and microbe–microbe interactions.

The composition of resident microbial communities is driven by nutrient availability (1–3), the physical environment (4, 5), host–microbiota interactions (6, 7), and interactions within the microbiota (8, 9). The sum of all these forces may shape the spatial arrangement of intestinal microbes, and, in turn, the spatial structure of the microbiota could influence how host–microbe and microbe–microbe interactions occur (10). The synergy between the microgeography of intestinal bacterial consortia and the interactions of microbes with their environment or other microbes has been studied in vitro using synthetic communities and computational simulations (11–15). In the context of the gastrointestinal system, studying the connection between the native spatial structure of the microbiota and its function naturally calls for three-dimensional (3D) imaging strategies that enable the simultaneous visualization of bacterial communities and host structures at multiple scales (16, 17). However, existing 3D imaging approaches remain hindered by the opacity of intestinal tissues and their contents as well as their impermeability to labeling probes. Methods have been developed to obtain cross-sectional slices from paraffin- or plastic-embedded intestinal tissues (18–20). Thin sections eliminate the optical and diffusion barriers that thick tissues present to imaging and molecular staining, but they fragment host tissues and microbial assemblies. The advent of tissue-clearing technologies has enabled the imaging of cellular structures in thick tissues such as the brain (21, 22). However, the full potential of tissue-clearing techniques has yet to be realized to quantify the composition and organization of the host–microbiota interface with spatial resolution.

Sequencing of bacterial 16S ribosomal RNA (rRNA) genes has been effective at surveying the composition of the bacterial microbiota in different compartments along and across the gastrointestinal tract (GIT). Indeed, sequencing has revealed that the mucosal microbiota is distinct and spatially heterogeneous, and bioinformatics tools have enabled the inference of bacterial networks of interaction (23–33). However, sequencing alone cannot be used to reconstruct the spatial distribution of bacteria relative to the host with high spatial resolution. Therefore, microscopic imaging of thin sections of intestinal tissue is the de facto approach to study the fine spatial structure of the microbiota and the host (2, 18, 19, 34). Thin-section imaging (TSI) is ordinarily coupled with fluorescence in situ hybridization (FISH), immunohistochemistry, and other labeling methods that link the molecular identity of bacteria and host elements to their location. For example, TSI has been used to study the spontaneous segregation of *Escherichia coli* and mucolytic bacteria in the colonic mucus layer (35), by measuring the distance of different bacterial taxa from the epithelial surface (19), such as during inflammation (36). In notable recent examples of the quantitative application of TSI, semiautomated computational image analysis was used to measure the thickness of the colonic mucus layer and the proximity of bacteria to the host as a function of diet (18), and highly multiplexed FISH was used to investigate the microscopic spatial structure of microbiota in the distal colon (20).

Although TSI is valuable to investigate the biogeography of the intestines and the microbiota, it is unable to completely capture the spatial structure of bacterial communities in the gut. The first limitation of TSI is that it sets 2D bounds on the spatial exploration of a heterogeneous, 3D system. TSI sections are typically 5 µm to 10 µm thick, whereas topographic epithelial features and mucosal microbial communities can be one to four orders of magnitude larger. Mucosal biofilms can be hundreds of microns long (37), and bacterial colonies in the colonic crypts have a heterogeneous taxonomic composition with a 3D spatial structure that cannot be charted unless the entire crypt (diameter 50 µm) is imaged (30, 38).

Quantitative descriptions of the 3D spatial structure of native bacterial biofilms with taxonomic resolution are challenging to develop because of the natural opacity of the intestinal tissue and contents, and the complex composition of the microbiota, in which potentially hundreds of bacterial species coexist. Moreover, a quantitative description of a diverse and spatially heterogeneous system requires abundant data that can only be obtained through unrestricted optical access to samples. Tissue-clearing techniques have been developed for some tissues and organs (including brain, heart, kidney, lung, stomach, and sputum) (22, 39–42). However, the direct application of tissue-clearing techniques typically results in the loss of the delicate mucus layer and associated bacterial communities (43). CLARITY (clear lipid-exchanged acrylamide-hybridized rigid imaging/immunostaining/in situ hybridization-compatible tissue hydrogel) and PACT (passive CLARITY technique) techniques involve multiple mechanically stressful sample-preparation steps to transform the cellular matrix of tissue into an acrylamide gel (21, 22, 40). Moreover, application of CLARITY or PACT to whole-mount tissues would irreversibly deform them and destroy the patterns of bacterial colonization on the mucosa.

Here, we developed an advanced tissue-clearing technique that preserved the spatial structure of the mucosal microbiota and the host tissue, including the delicate mucus layer. We combined this method with sequencing of 16S rRNA genes, amplified in situ labeling of rRNA, spectral imaging, and statistical analyses. This method is capable of revealing patterns in the composition of the microbiota with taxonomic and spatial resolution. We used this methodology to test the effects of antibiotic on the bacterial colonization of the intestinal mucosa. We were able to quantify patterns in the spatial structure of the mucosal microbiota of the cecum at multiple scales and at different stages of antibiotic exposure.

## Results

### Sample Preparation, Staining, and Imaging.

**Fig. 1.**
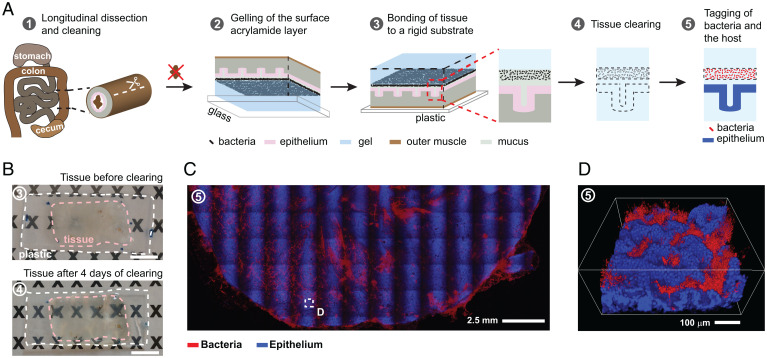
Sample preparation and imaging for 3D mapping of the mucosal microbiota’s spatial structure. (*A*) The workflow of the method has five key steps in which a section of intestinal tissue is prepared for whole-mount confocal imaging of the mucosal microbiota. (*B*) A sample of preserved murine cecal tissue before and after 4 d of lipid removal. The dimensions and shape of the sample were not visibly altered by clearing. (Scale bars: 1 cm.) (*C*) Maximum intensity projection of a tiled image of a typical intestinal tissue sample after the method. The image of the cecum was obtained by stitching multiple fields of view acquired with a 5× objective that is not flat-field corrected. Bacteria were stained by HCR with a eubacterial detection probe, and host nuclei were stained with DAPI. (*D*) The 3D rendering of the confocal imaging of the area enclosed in the dashed white square in *C* shows the location of bacteria with respect to each other and the host.

To achieve unrestricted optical access to the mucosa, we developed a tissue clarification method that exposes the intestinal mucosa in a fully laid out display (Fig. 1). Mounting tissue samples flat enabled us to image any point of the mucosa using a standard confocal microscope, and clearing the tissue increased the depth of imaging with refractive index–matching long-working-distance objectives (*SI Appendix*, *Supplementary Materials and Methods*). However, to achieve optical transparency of exposed intestinal tissues, we had to solve multiple experimental challenges.

To maintain the spatial integrity of bacteria and mucus during whole-mount sample preparation, we developed a method that addresses the preservation of the materials on the tissue surface separately from the preservation of the rest of the sample, and that minimizes the duration of steps that can dislodge mucus and biofilms. The overall workflow of our method (Fig. 1*A*), which we developed in a murine model, was as follows: After careful dissection and removal of intestinal contents, tissues were fixed in paraformaldehyde for 1 h to prevent biochemical decay. Next, we created a capillary layer of acrylamide mix between the exposed mucosa and the glass bottom of a shallow chamber. Upon heating, the acrylamide mix polymerized into a surface gel layer with a thickness on the order of 100 μm. Once the mucosal surface of the sample was protected, the remainder of the tissue was embedded and gelled. Finally, the uncovered surface of the sample (the muscle side) was glued to a rigid, flat, plastic substrate to keep the sample flat (Fig. 1*B*). In this configuration, samples could be passively cleared, stained, and imaged without damaging the mucosal surface. A detailed description of the workflow is available here (*Materials and Methods*).

To locate bacteria in situ, we fluorescently labeled bacterial 16S rRNA transcripts through hybridization chain reaction (HCR) (44, 45) (*Materials and Methods*). Standard FISH probes are labeled with up to two fluorophores, which produce a fluorescent emission that is sufficiently intense to image bacteria on thin sections. However, bacteria in the mammalian gut can be found in thick biofilms, epithelial crypts, or across the epithelial barrier, all of which obscure visibility. Therefore, we used HCR for labeling because it increases the intensity of fluorescence by at least one order of magnitude compared with FISH probes (44).

The method presented here enables the mapping of bacteria on the mucosa at multiple length scales. To reveal patterns of colonization over spatial scales on the order of centimeters, tissue samples were imaged in a laser-scanning confocal microscope at low magnification (5×), and the images were tiled (Fig. 1*C*). To image the detailed spatial structure of bacterial biofilms with micrometer resolution (Fig. 1*D* and Movie S1), we mounted samples in a refractive index–matching solution (RIMS; *n* = 1.46) and used a 20× CLARITY objective with a collar for the compensation of spherical aberrations (*Materials and Methods*).

### Sensitivity and Specificity of Bacterial Staining.

**Fig. 2.**
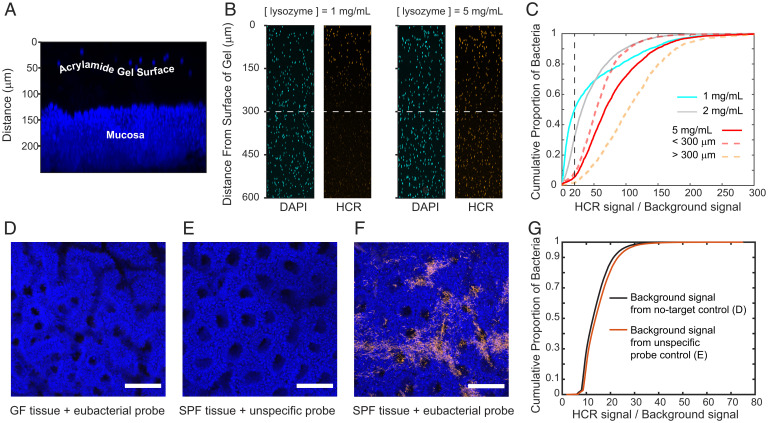
Sensitivity and specificity of fluorescence imaging of bacteria embedded in acrylamide gels using dual embedding. (*A*) Maximum intensity projection of a digital cross-section (152 µm) of intestinal tissue. The thickness of the protective acrylamide gel layer was revealed by blue-fluorescent beads on its surface. The layer of gel was a diffusive barrier for lysozyme during HCR staining of bacteria. (*B*) Maximum intensity projections of digital cross-sections (50 µm) of gel slabs seeded with bacteria. The effect of lysozyme concentration on the sensitivity of HCR staining is illustrated. At a suboptimal concentration of lysozyme (1 mg/mL), only bacteria near the surface of the gel could be detected, whereas a concentration of lysozyme of 5 mg/mL enabled the detection of bacteria throughout the gel. (*C*) Experimental cumulative distributions of HCR staining of bacteria embedded in gel slabs that were treated with different lysozyme concentrations. At a lysozyme concentration of 5 mg/mL, ∼94% of bacteria within 600 µm of the surface had an HCR signal-to-background signal ratio of ≥20 (vertical dashed line). (*D*–*F*) Maximal intensity projections of representative luminal views of proximal colon tissue that was used to test the specificity of HCR staining of bacteria in situ. (*D*) HCR with a eubacterial detection sequence (eub338) on GF tissue, (*E*) HCR with a nonspecific control probe (non338) on tissue from mice with a microbiota (SPF), and (*F*) HCR with a eubacterial detection sequence (eub338) on tissue from mice with a microbiota. (Scale bars: 100 µm.) (*G*) Experimental cumulative distribution of the HCR signal-to-background signal ratio from controls for in situ HCR staining of bacteria in *D*–*F*. Three fields (*n* = 3) of view from each sample (*D*–*F*) were acquired. The average intensity of the background signal was calculated from the controls with no target and a nonspecific probe. In *F*, bacteria were segmented with an intensity filter to obtain their average HCR fluorescence.

Sensitive and specific identification of mucosal bacteria through fluorescence imaging was accomplished by optimizing HCR tagging and controlling for off-target effects (*Materials and Methods* and *SI Appendix*, *Supplementary Materials and Methods* and Figs. S1–S4 and S6–S8). Fluorescent tagging through HCR was achieved by making the bacterial cell wall permeable to DNA probes and HCR hairpins. However, the acrylamide gel sheet that we created to protect the mucosal surface of samples formed a barrier for the diffusion of lysozyme (Fig. 2*A*) that digests the bacterial peptidoglycan. Poor permeabilization of bacteria limits the sensitivity of imaging to bacteria closer to the mucosal surface and impedes the detection of bacteria deep in the tissue samples. To determine the correct concentration of lysozyme for optimal permeabilization of the cell wall, we created acrylamide gel slabs and embedded them with gram-positive (*Clostridium scindens*) and gram-negative (*Bacteroides fragilis*) bacteria. The purpose of these gels was to mimic the geometry and composition of the acrylamide layer on tissue samples. The gel slabs were obtained by using the same procedure as in the preservation and clearing of tissues, had similar dimensions to tissue samples, and were exposed to lysozyme on one side only (*SI Appendix*, *Supplementary Materials and Methods* and Figs. S1 and S2). The duration of the treatment with lysozyme was kept constant at 6 h, and we varied the concentration of lysozyme in the range 1 mg/mL to 5 mg/mL to determine the optimal concentration for bacterial permeabilization. Bacteria were tagged with an HCR probe that included a eubacterial detection sequence (eub338), and we imaged from the surface of the gels to a depth of 600 µm (Fig. 2*B*). We measured the intensity of HCR tagging of bacteria, which were identified with the blue-fluorescent DNA intercalated dye DAPI. The sensitivity of our method was defined as the proportion of bacteria down to 600 µm with a fluorescent signal-to-background ratio of ≥20 (Fig. 2*C*). At a lysozyme concentration of 5 mg/mL, sensitivity was 94%, and it dropped to ∼50% for 1 mg/mL.

Nonspecific detection and amplification are potential sources of background signal in HCR. Control experiments showed that, in the absence of a target (germ-free [GF] + eub338) or a detecting probe (specific-pathogen-free [SPF] + non338), there was no amplification, whereas, when both the target and the probe were present (SPF + eub338), there was amplification (Fig. 2 *D*–*F*) (*SI Appendix*, *Supplementary Materials and Methods*). Plotting the intensity values showed that in situ HCR tagging of bacteria produced a signal that is 8.5 to 9 times as strong as the background in 90% of bacteria (Fig. 2*G*).

### General 3D Spatial Organization of Bacteria in the IIeum, Cecum, and Proximal Colon.

To evaluate our 3D imaging methods, we imaged bacteria, mucus, and the host epithelium in disparate sections of the GIT with different biological functions, mucosal topographies, and amounts of mucosal materials (46, 47).

#### Proximal colon.

**Fig. 3.**
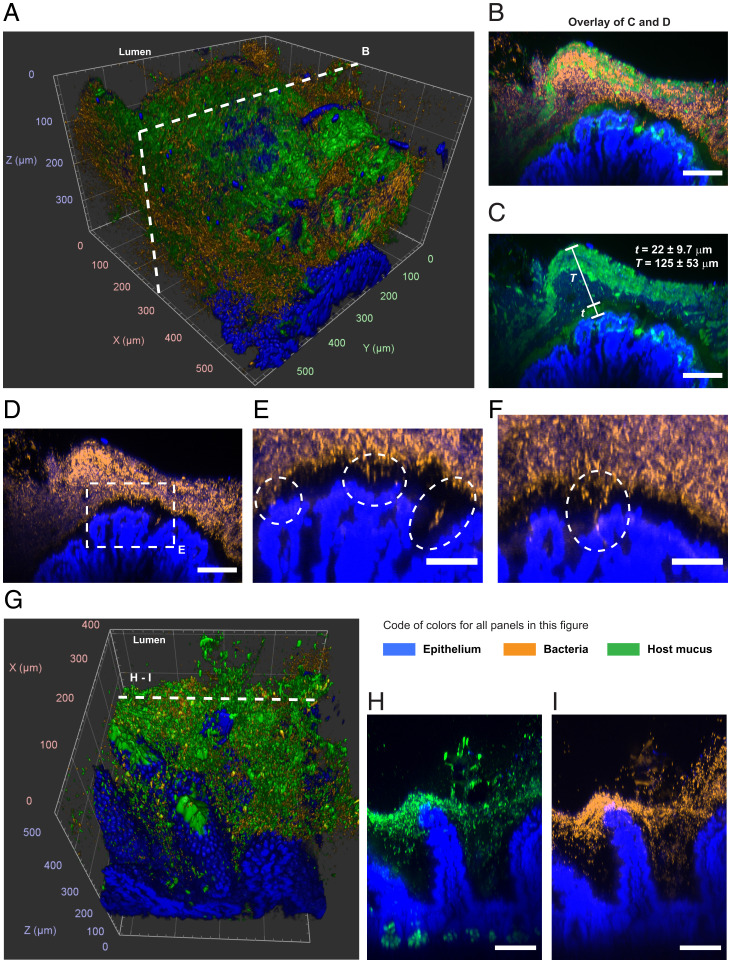
Spatial structure of the host–microbiota interface of the murine proximal colon and distal ileum after being processed with the method presented here (Fig. 1). (*A*) The 3D rendering of confocal imaging (20×) of the crest of a fold in the proximal colon. The epithelium (blue) was covered by a mix of mucus (green) and bacteria (orange). (*B*–*D*) Maximum intensity projection of the digital cross-section (7 µm) depicted in *A*. Mucus and bacteria were organized in well-defined layers. Two layers of mucus separated most of bacteria from the mucosa and from the luminal contents (removed from this area of the sample). The thin layer of mucus that separated the epithelium from the majority of the microbiota in the lumen could be crossed by bacteria in healthy tissue. (*E*) Zoom-in view from *D*. Ovals are examples of bacteria inside and across the thin mucus layer that lines the epithelium. (*F*) Maximum intensity projection of a digital cross-section (7 µm) from the same sample as in *A*. Inside the oval is another example of bacteria crossing the thin mucus layer and the epithelium. (*G*) The 3D rendering of confocal imaging (20×) of villi of the small intestine covered with mucus and bacteria. (*H* and *I*) Maximum intensity projections of the digital cross-section (16 µm) depicted in *G*. Bacteria accumulated on mucus around the top of villi. (All scale bars: 100 µm.)

At low magnification (5×), we observed the crests and valleys of the epithelial folds and that most of the mucosa was covered by food particles and mucus (*SI Appendix*, Fig. S5*a*). At higher magnification (20×), our method enabled the exploration of the 3D organization of the host–microbiota interface in the proximal colon (Fig. 3*A* and *SI Appendix*, Fig. S9). The 3D imaging can be analyzed through digital cross-sections with arbitrary orientation and thickness. Examining digital cross-sections, we found that bacteria were mixed with mucus threads and granules in a layer that had an average thickness of 125 µm (Fig. 3 *B and* *C* and Movie S2). We also found that bacteria were separated from the epithelium by a single layer of mucus with an average thickness of 22 µm. The 3D imaging provides the ability to examine tissues in their totality through computational 3D rendering. Thus, we were we able to scan the tissue and find rare but conspicuous locations where bacteria had penetrated the mucus layer or crossed it and reached a crypt and the subepithelial space (Fig. 3 *D–F*).

#### Ileum.

At low magnification (5×), imaging revealed that bacteria were not uniformly distributed throughout villi and were mostly found as part of large agglomerations of food particles and mucus that adhere to the epithelium (*SI Appendix*, Fig. S5*b*). At higher magnification (20×), 3D imaging showed that bacteria were contained by mucus to a layer near the top of villi (Fig. 3 *E* and *F*).

#### Cecum.

**Fig. 4.**
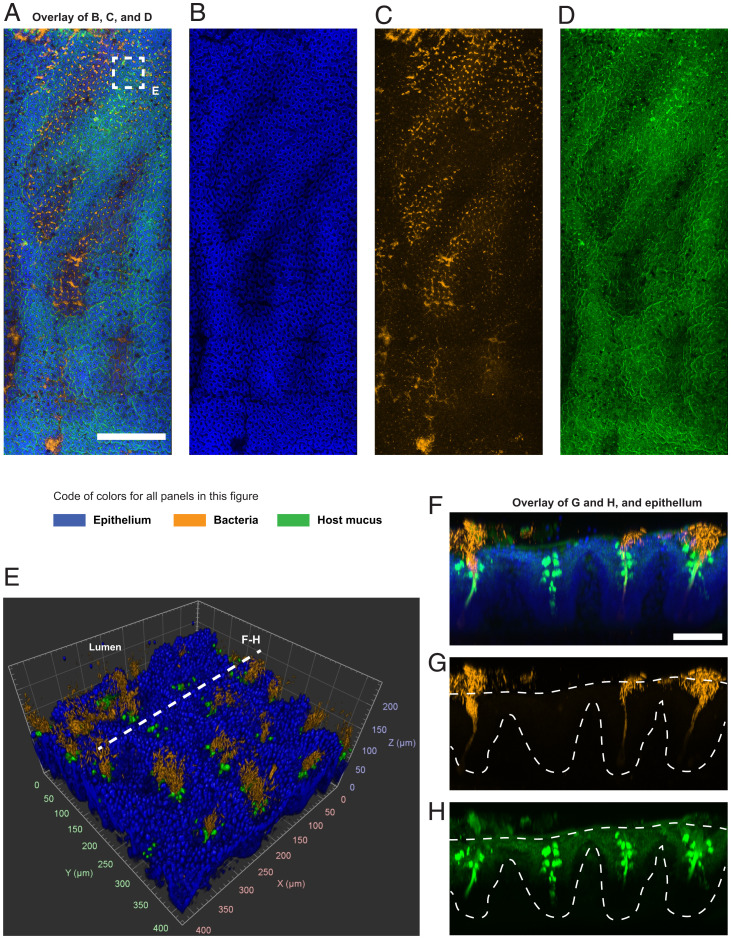
Multiscale imaging showed that the cecal mucosa was colonized in clusters. (*A*–*D*) Tiled image of luminal imaging of a tissue sample from the cecum. The image was obtained by stitching multiple fields of view acquired at 5× magnification. Bacteria were stained by HCR with a eubacterial detection probe (orange), the DNA of host cells was stained with DAPI (blue), and the host mucus was stained with WGA lectin (green). The epithelium of the cecum was lined with crypts, some of which were isolated and some of which were connected to other crypts by crevices. The colonization of the mucosal crypts was discontinuous. Clusters of colonized crypts were separated by areas with fewer bacteria. The spatial distribution of mucus was more uniform. (Scale bar: 1 mm.) (*E*) The 3D rendering of confocal imaging (20×) of the cecal mucosa enclosed in the dashed square area in *A*. (*F*–*H*) Maximum intensity projection of the digital cross-section (70 µm) is indicated by a dashed line in *E*. Dashed lines in *G* and *H* indicate the approximate location of crypts in *F*. Bacteria that colonized the cecum occupied the crypts and the mucus these glands secrete. All crypts produced mucus, but not all crypts were colonized by bacteria. (Scale bar: 75 µm.)

The epithelial layer of the murine cecum is organized as a regular array of recessed mucus-secreting glands known as crypts (48). At low magnification (5×), imaging showed that bacteria in the cecal mucosa formed colonies that were associated with one or multiple crypts (Fig. 4 *A–D*). However, the colonization of crypts was not homogeneous across the tissue. Colonized crypts were spatially clustered and surrounded by crypts with few or no bacteria. In contrast, mucus was somewhat evenly distributed across crypts. The 3D imaging at higher magnification (20×) confirmed that not all crypts were occupied by bacterial colonies, but that all crypts secreted mucus (Fig. 4* E–H*).

### Quantification of the Composition and Spatial Structure of the Microbiota of Crypts.

As shown in our 3D imaging of the mucosa (Figs. 3 and 4), bacteria occupy habitats with different geometries along the mouse GIT. In the proximal colon, bacteria accumulated in a layer that ran parallel to the epithelium, whereas, in the cecum, bacteria were split into colonies that were associated with crypts. The microbiota of the cecal mucosa and of intestinal crypts are diverse (30). However, the spatial structure of these communities remains unexplored.

**Fig. 5.**
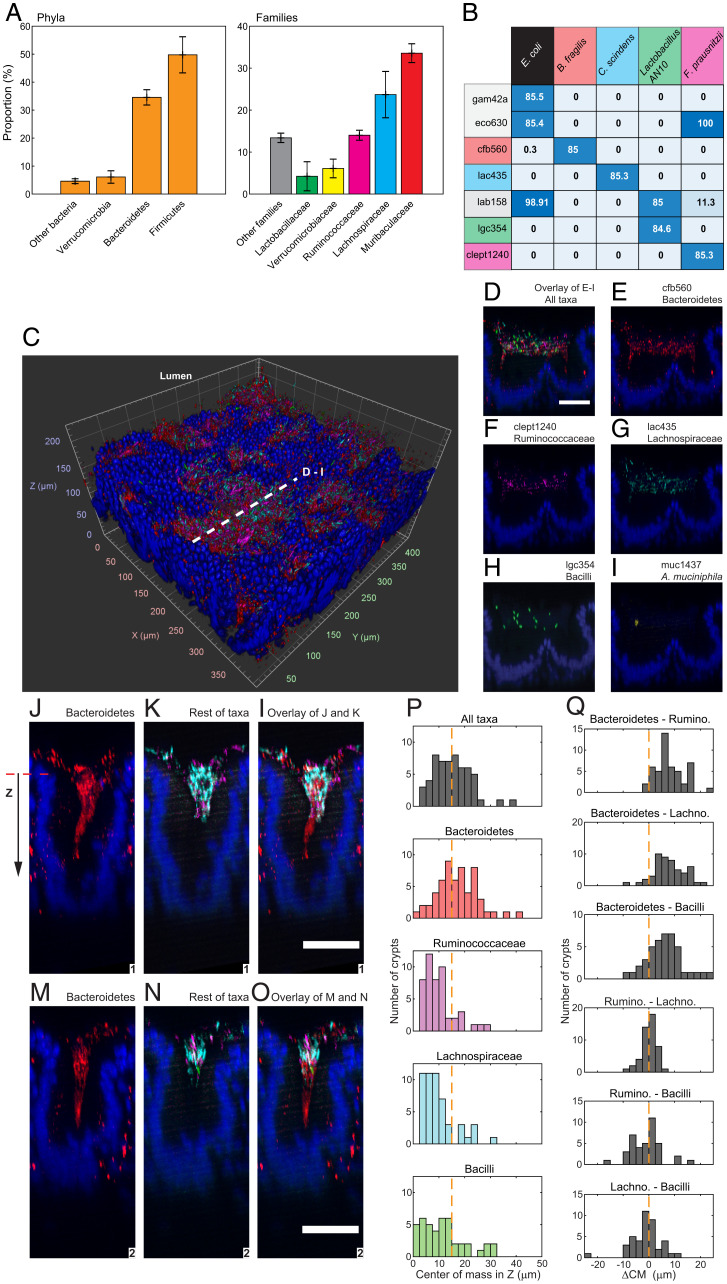
Multiplexed staining of the native mucosal microbiota was specific and revealed the structure of colonies within crypts. (*A*) Taxonomic composition of the bacterial microbiota of the cecum according to the sequencing of the 16S rRNA gene. In both plots, each bar represents the mean proportion of a taxonomic group. Murine cecal mucosa for total DNA extraction was harvested from four mice (*n* = 4). Error bars represent the SD. (*B*) A matrix of bacterial taxa and detection sequences where each matrix element gives the percentage of bacterial cells experimentally tagged by HCR probe with each detection sequence. Ideally, a probe only hybridizes bacteria with perfectly homologous rRNA transcripts. Perfectly matching probe–taxon pairs (PMPs) are color coded, for example, lac435–*C. scindens*. We set a minimal detection threshold of 84% for PMPs. At this threshold, off-target HCR tagging was maximally reduced, and detection sensitivity was maximized. (*C*) The 3D rendering of cecal mucosa imaged at 20× magnification. Bacterial 16S rRNA on the sample was stained with multiple HCR probes with detection sequences cfb560, lac435, lgc354, clept1240, and muc1437. (*D*–*I*) Maximum intensity projection of the digital cross-section (5 µm) that is depicted in *C* with a dashed line. Multiplexed staining with the probes tested in *B* revealed the location of five taxonomic groups in a densely populated dual crypt. Nuclei of the epithelial layer of the crypt are colored in blue. (Scale bar: 50 µm.) (*J*–*O*) Maximal intensity projections of the digital cross-sections (10 µm) of two representative cecal crypts. Four taxa were imaged: Bacteroidetes (red), Ruminococcaceae (magenta), Lachnospiraceae (cyan), and Bacilli (green). Nuclei of the epithelial layer of the crypt are colored in blue. Bacteroidetes spanned the length of each colony, whereas Firmicutes remained near the luminal end of crypts that was used as the spatial reference in our analysis. Control experiments showed that the red fluorescent signal outside the crypts was an artifact of staining and was not included in the analysis. (Scale bars: 50 µm.) (*P*) Distributions of the center of mass of taxa over the ensemble of crypts. Because each taxon was not found in every crypt, the number of crypts in each distribution was different (*n* = 57 [all taxa], 57, 48, 51, and 43). Most Firmicutes were found between the median center of mass and the luminal end of the crypt, whereas Bacteroidetes dominated the space between the median and the bottom of crypts. (*Q*) Relative distance between the centers of mass (ΔCM) of bacterial taxa within single crypts. Firmicutes were centered around each other, while Bacteroidetes segregated from Firmicutes. ΔC was measured as the distance between the centers of mass of bacterial aggregates. If the distribution was centered around ΔC = 0, the pair of taxa colocalized inside the crypt. If ΔC was skewed, it means the two taxa segregated from each other within crypts. Nuclei of the epithelial layer of the crypt are colored in blue.

To explore the spatial order in the microbiota of cecal crypts, we extended our imaging method to enable multiplexed imaging of bacterial targets (*Materials and Methods* and *SI Appendix*, *Supplementary Materials and Methods*). First, to identify the taxa we should target for imaging, we sequenced the 16S rRNA gene of the microbiota of the cecum (Fig. 5*A*), and searched the literature for FISH probes that could specifically detect bacteria belonging to the five taxonomic groups that comprised ∼76% of the sequenced reads: Bacteroidetes, Lactobacillaeae, Ruminoccocaceae, Lachnospiraceae, and Verrucomicrobiaceae (Fig. 5*A*). We tested, in vitro, the sensitivity and specificity of the selected detection sequences in HCR (*Materials and Methods* and *SI Appendix*, *Supplementary Materials and Methods* and Figs. S3 and S4). We performed HCR with taxon-specific probes, targeting four species of bacteria that were representative of the target taxonomic groups. We used an additional probe for *E. coli* because it was not found in the sequencing of the cecal mucosa and thus served as a further control for the specificity of our probes (*SI Appendix*, Table S1). Finally, HCR probes for multiplex in situ imaging were designed by pairing a unique HCR hairpin pair to each detection sequence that detected at least 84% of its ideal target bacterium while being insensitive to the rest of the bacterial targets, with the exception of the detection sequence cfb560 that cross-reacts with 0.3% of *E. coli* targets (Fig. 5*B*). The promiscuous lab158 probe was rejected in favor of the orthogonal lgc354 suite.

Because we had observed that cecal crypts are colonized in patches (Fig. 4*C*), we performed multiplexed HCR on several cecum samples and imaged the most abundant target taxon (Bacteroidetes) at low resolution (5×) (not shown) to locate patches.

Within one patch of crypts, we obtained spectral imaging at higher magnification (20×), which was processed computationally to remove the fluorescent spectral overlap (*SI Appendix*, *Supplementary Materials and Methods*). The 3D spectral imaging with linear deconvolution of the cecal mucosa clearly showed multispecies colonization (Fig. 5*C* and Movie S3), and distinguished the location of different taxa in dense cryptal colonies (Fig. 5 *D*–*I*). We analyzed the taxonomic composition of a subset of 57 abundantly colonized crypts using commercial 3D image analysis software (*Materials and Methods* and Movie S4). We measured the abundance (number of voxels) and the position of the target taxa inside crypts. Accordingly, the crypt microbiota was 65% Bacteroidetes, 18% Lachnospiraceae, 13% Ruminococcaceae, and 3% Bacilli, with an insignificant proportion of *Akkermansia*. Also, in this small set of crypts, the taxa were arranged in different depths within each crypt, with Bacilli, Lachnospiraceae, and Ruminococcaceae found closer to the luminal end (Fig. 5 *J*–*Q*).

### The Biogeography of the Mucosal Microbiota Is Robust to Taxonomic Changes Driven by Ciprofloxacin.

**Fig. 6.**
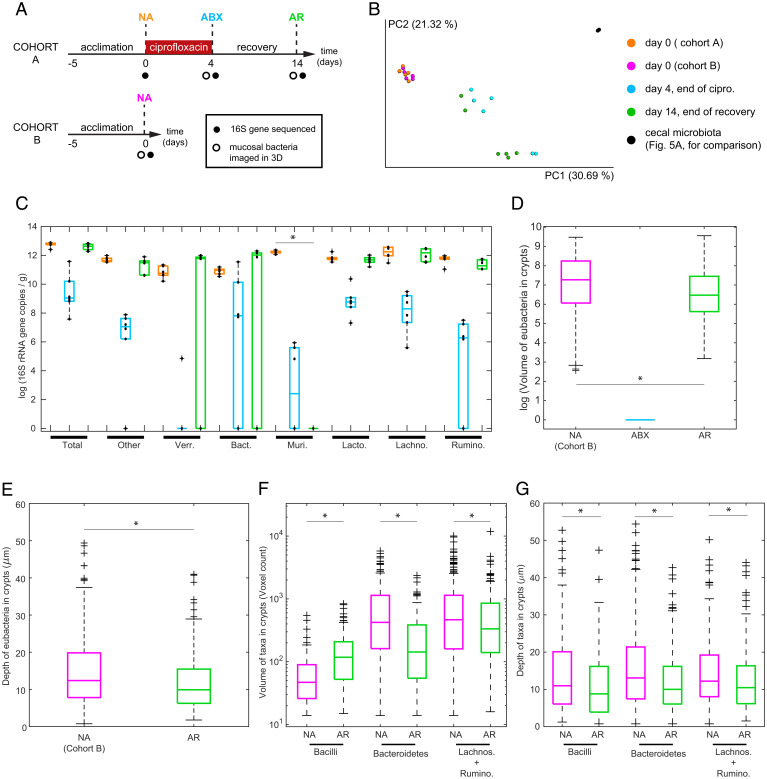
Quantitative sequencing of the fecal microbiota and 3D biogeography of bacteria in cecal crypts from 3D imaging at different stages of the antibiotic challenge. (*A*) Overview of the experimental design for the antibiotic challenge. In cohort A, the antibiotic was administered for 4 d, after which the microbiota was allowed to recover for 10 d. Cohort B was not given antibiotic. Mice in cohort B and cohort A had a similar microbiota at day 0 (*B*). (*B*) A principal coordinates analysis plot was created using the matrix of paired-wise distance between samples calculated using the Bray–Curtis dissimilarity with amplicon sequence variants from 16S rRNA gene sequencing (*SI Appendix*, *Supplementary Materials and Methods*). Fecal microbiota of mice after the acclimation period were similar in both cohorts (cohort A in orange and cohort B in magenta). The composition of the microbiota diverged between cages after the treatment with ciprofloxacin (blue), and differences remained 10 d after the antibiotic was removed (green). Cecal microbiota from four untreated mice shown previously in Fig. 5*A* is shown for comparison (black). (*C*) Total abundance of bacteria and of bacterial families in feces obtained by quantitative sequencing; no ciprofloxacin treatment (orange), after 4 d of ciprofloxacin (blue), and at the end of the 10-d period of recovery (cohort A, green). The abundance of bacteria was measured by the number of copies of the 16S rRNA gene per unit mass of feces. Data points are overlaid onto box plots (*n* = 6 mice in three cages). On each box, the central line indicates the median, and the bottom and top edges of the box indicate the 25th and 75th percentiles, respectively. The whiskers extend to the minimum and maximum values not considered outliers, with outliers beyond. Bacterial families are only shown if they were present in at least four out of six mice. Families that were not imaged, for lack of a specific probe, were lumped (“Other”). After the 10-d recovery, Muribaculaceae were not detected in any of the six sequenced mice (*P* = 0.0022 at 5% significance, Wilcoxon rank sum test, *P* = 0.0154 after Benjamini–Hochberg significance at 0.25 false discovery rate). Taxon abbreviations in panel *C*: Verr, Verrucomicrobiaceae; Bact, Bacteroidaceae; Muri, Muribaculaceae; Lacto, Lactobacillaceae; Lachno, Lachnospiraceae; Rumino, Ruminococcaceae. (*D*–*G*) Biogeography of bacteria from 3D imaging. (*D* and *E*) Distributions of the volume (voxel counts) and depth of bacteria in crypts from mice at different stages of exposure to ciprofloxacin. Box plots are defined as in *C*. The difference in depth and volume between the untreated and recovered mice was statistically significant according to a Wilcoxon rank sum test (*P* < 0.001 at 1% significance). (*F* and *G*) Distributions of the volume and depth of bacterial taxa Bacilli, *Bacteroidetes*, and combined Clostridiales (Lachnospiraceae and Ruminococcaceae) in crypts from mice not exposed to ciprofloxacin (NA, no antibiotic) and in crypts that were recolonized after the antibiotic was removed for 10 d (AR, after recovery). Data are from analysis of 468 crypts from five mice (two controls and three treated). Significance was determined by Wilcoxon rank sum test at 5% significance followed by a Benjamini–Hochberg correction for multiple comparisons at 0.1 false discovery rate. In *F* and *G*, * denote the test was significant after the Benjamini–Hochberg correction. In *C* and *D*, data with value zero were substituted with value one to enable the calculation of the logarithm.

To investigate the robustness of clustered crypt colonization and the particular role of Muribaculaceae (formerly S24-7 family) on the colonization of crypts, we used the broad-spectrum antibiotic ciprofloxacin. Recently, it was shown that Muribaculaceae can become undetectable in the feces of conventional mice up to 10 d after stopping a 5-d treatment with ciprofloxacin, whereas other taxa seem to recover to their initial numbers earlier (49). Accordingly, we administered 4 mg of ciprofloxacin twice per day for 4 d[Sec s11] and allowed the microbiota to recover for 10 d (Fig. 6*A*).

The effect of ciprofloxacin on the composition and spatial organization of the mucosal microbiota was investigated through quantitative sequencing of the 16S rRNA gene of the fecal microbiota (Fig. 6 *B* and *C*) and 3D imaging (Fig. 6 *D*–*G*) of the cecal crypts of two sets of mice. Mice of cohorts A and B were of the same age and from the same room at the facility of origin, and they had a similar fecal microbiota composition before the administration of ciprofloxacin (Fig. 6*B* and *Materials and Methods*). After 4 d of twice-daily administration of oral ciprofloxacin, we imaged the microbiota of the cecal mucosa of three mice from three separate cages (cohort A). The remaining mice from each cage were given a 10-d-long postantibiotic recovery period. After this period, we imaged the microbiota of the cecal mucosa of another set of three mice from three separate cages (cohort A). To control for the effects of antibiotic, we imaged the microbiota of the cecal mucosa of two control mice that were not exposed to the drug (cohort B).

We quantified the absolute total abundance of bacteria in feces through qPCR and then quantified the absolute abundance of individual bacterial families by multiplying the absolute total abundance by the proportions obtained from the sequencing of 16S rRNA gene (50) (*Materials and Methods* and *SI Appendix*, Tables S3 and S4) (Fig. 6*C*). The median of the total bacterial load in feces was reduced by ciprofloxacin by more than three orders of magnitude among the three cages of cohort A (*n* = 6) (Fig. 6*C*, Total, blue). Ten days after discontinuing the antibiotic, the average bacterial load in feces recovered to the same order of magnitude as the preantibiotic abundance (*n* = 6) (Fig. 6*C*, Total, green).

We found that the change in the abundance of bacteria after recovery from ciprofloxacin was not uniform across taxa and the cages of cohort A (Fig. 6*C*). As expected, the family Muribaculaceae was undetected in all recovered mice (*SI Appendix*, *Supplementary Materials and Methods* and Tables S3 and S4 ). Compared with the control mice not treated with ciprofloxacin, the absolute abundance of *Bacteroides,* Verrucomicrobia, Ruminococcaceae, Lactobacillaceae, and other families were not statistically significantly different after 10 d of recovery. The dominant *Bacteroides* species in two of the three cages after recovery was *Bacteroides thetaiotaomicron*, whereas, in the third cage, no Bacteroidetes was detected (*SI Appendix*, *Supplementary Materials and Methods* and Table S4).

Four days of twice-daily ciprofloxacin reduced the size of bacterial colonies in crypts to zero (Fig. 6*D*), but, after 10 d without antibiotics, bacterial colonies grew to a size comparable to colonies that were unexposed to ciprofloxacin (*SI Appendix*, *Supplementary Materials and Methods* and Figs. S10–S12). To quantify the recovery of the mucosal microbiota after ciprofloxacin, we compared the spatial distribution of bacteria of crypts in unexposed (no ciprofloxacin) and recovered (10 d after withdrawing ciprofloxacin) mice.

At the level of single crypts, we compared the abundance of bacteria (volume) and their proximity to the host (depth) in the crypts of control mice (no antibiotic) and the crypts of mice that had recovered from antibiotic for 10 d (*SI Appendix*, Figs. S10–S12). The volume of bacterial colonies was measured as the voxel count of the objects segmented in the eubacterial channel (*Materials and Methods* and *SI Appendix*) (Fig. 6*D*), and the depth of bacteria was measured as the relative position of their center of mass with respect to the luminal opening of crypts (Fig. 6*E*). We found that, although bacteria recolonized crypts after a 10-d recovery period, the volume and depth of crypt colonies were both less than the volume and depth of crypt colonies in the mice that were not exposed to ciprofloxacin[Sec s11]. The median volume of eubacteria in recolonized crypts was less than half of the median volume of unexposed colonies (45%), and the median position of eubacteria in unexposed crypts was 2.5 μm deeper. Images of the bacterial colonies of cecal crypts (*SI Appendix*, Figs. S10–S12) illustrate the varied colonization of crypts.

In unexposed and recovered mice, we measured the spatial distribution of three taxonomic groups across and within single crypts: Bacilli, Bacteroidetes, and Clostridiales. Within Clostridiales, we considered the closely related Lachnospiraceae and Ruminococcaceae, whose HCR probes were labeled with the same fluorophore (A594). We measured the volume (voxel count) and depth (Fig. 6 *D*–*G*), and calculated the relative distance of taxa to eubacteria within crypts (*SI Appendix*, Fig. S24). Although total abundance of bacteria was similar between recovery and unexposed samples (Fig. 6*D*), the median abundance of Bacilli was larger in the crypts of mice after recovery than in unexposed mice. Across taxa, the center of mass of bacteria in crypts was significantly closer to the luminal opening in recovered mice compared with unexposed mice (Fig. 6*G*). We also found that Bacilli were the closest taxonomic group to the lumen (*SI Appendix*, Fig. S24), whereas Clostridia and Bacteroidetes were always the deepest colonizers. We also optimized the hybridization of rRNA of *Akkermansia muciniphila* to the taxon-specific muc1347 probe (*SI Appendix*, *Supplementary Materials and Methods*). Unlike the other taxa, we only found one big cluster of *A. muciniphila* within cecal crypts (*SI Appendix*, Fig. S22).

**Fig. 7.**
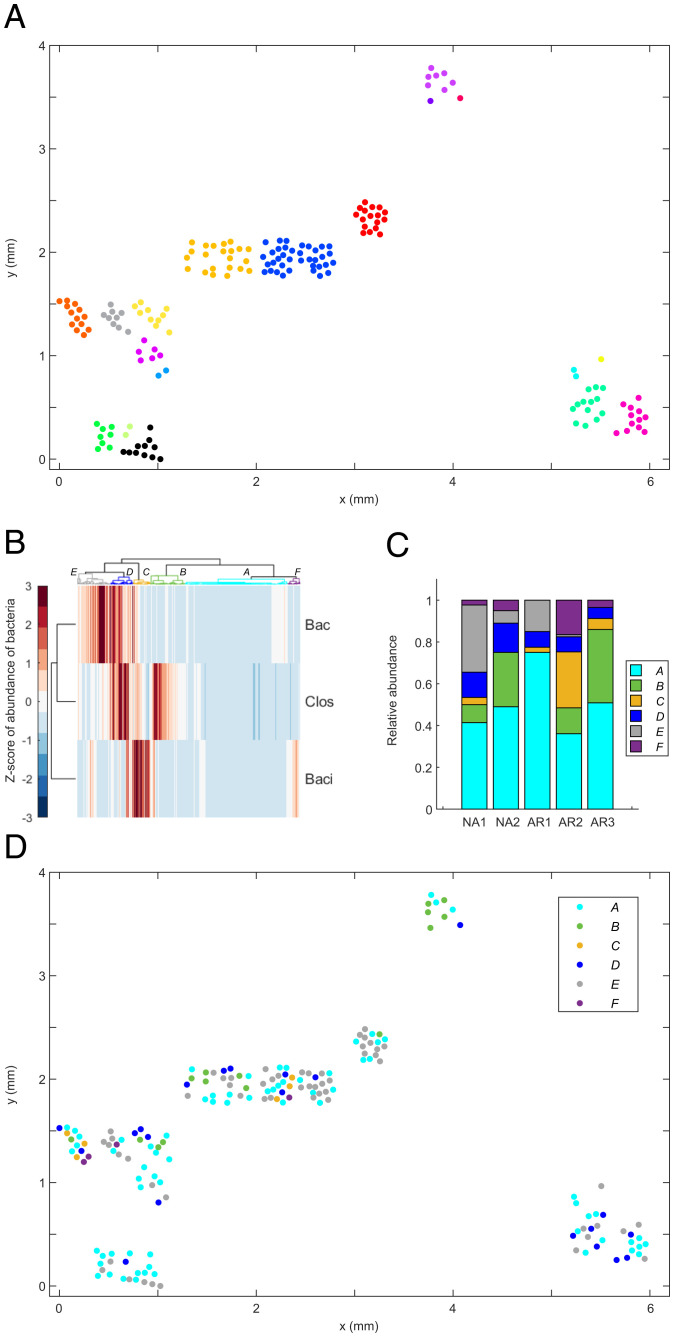
Multiplexed 3D imaging of the mucosal microbiota enabled the biogeographical analysis of bacterial colonies in murine cecal crypts according to their taxonomic composition. (*A*) Map of the location of crypts that were colonized by bacteria (colored dots) on the cecal mucosa of one mouse that was not exposed to ciprofloxacin (cohort B). Clusters of bacterially colonized crypts were identified computationally based on the distance between them. Any two crypts were considered as part of the same cluster if the distance between their center of mass on the (*x*, *y*) plane was less than or equal to 150 µm, which is approximately 2 times the typical distance between the center of contiguous crypts. Crypts within the same cluster were given the same color. (*B*) Heat map and clustering tree for the HCA of crypt communities (horizontal axis) in untreated and recovered mice based on the Z-scored abundance (voxel count) of three taxonomic groups (Bacteroidetes (“Bac”), Clostridia (“Clos”), and Bacilli (“Baci”)). A total of 468 single-crypt communities are represented here (274 from unexposed mice, 194 from recovery mice). (*C*) Relative abundance of crypt community types in two mice that were not exposed to ciprofloxacin (NA) and three mice whose microbiota was disturbed with oral ciprofloxacin for 4 d and then allowed to recover from 10 d (AR). (*D*) Map of the location of bacterial communities within crypts according to their taxonomic makeup (type), on the cecal mucosa of one mouse (NA1 in *C*) that was not exposed to ciprofloxacin (cohort B). Analogous plots as in *A* and *C* are available for another unexposed sample and three samples at day 14 (*SI Appendix*, Figs. S13, S14, and S16–S19).

At the scale of tissue samples, we measured the density of colonized crypts by counting the number of colonized crypts per imaged field of view (at 20×), which constitutes a surface area of 425 × 425 μm^2^ and can generally enable visualization of ∼20 mouse cecal crypts. A total of 296 and 199 colonized crypts were counted in the unexposed and recovered mice, respectively. We found that the mean density was of 9.3 ± 4.6 occupied crypts per field of view in untreated mice, whereas, after recovery, the mean density dropped to 5.4 ± 3.4 occupied crypts per field of view (*SI Appendix*, Fig. S25). Next, we mapped the location of crypts on the plane of the tissue and identified, computationally, the clusters of crypts (*Materials and Methods*, Fig. 7*A* and *SI Appendix*, Figs. S13 and S14). We found that the median number of crypts in a cluster in unexposed mice was four (75% of crypts in clusters between 1 and 7.5 crypts), whereas crypts that were recolonized after withdrawing ciprofloxacin formed clusters with a median size of two (75% of crypts were in clusters between one and four crypts).

To investigate the effect of ciprofloxacin on the distribution of bacterial taxa across the cecal mucosa, we first used a hierarchical clustering analysis (HCA) to classify crypt colonies according to their taxonomic makeup (Fig. 7); then we mapped the classification to the physical space. In HCA, bacterial colonies in single crypts were classified and grouped by the Z score of the abundance of Bacilli, Bacteroidetes, and Clostridia. The Z-scored abundance of a taxon quantifies its enrichment with respect to the mean in each crypt. The HCA’s classification tree and heatmap showed that crypt colonies could be binned into approximately six types (noted as A through F). Crypts in type A (cyan) were not enriched in any taxon, whereas crypts in type B were mostly enriched in Clostridia, crypts in type C and F were mostly enriched in Bacilli, crypts in type E were mostly enriched in Bacteroidetes, and crypts in type D were enriched in both Bacteroidetes and Clostridia. To verify the HCA classification visually, we used a T-distributed Stochastic Neighbor Embedding (t-SNE) and Uniform Manifold Approximation and Projection for Dimension Reduction (UMAP) dimensionality reductions of the Z-scored taxonomic abundances. These algorithms do not identify clusters but are designed to preserve the neighborhood of data points (*SI Appendix*, Fig. S15). Also, we calculated the silhouette score of the HCA to evaluate the quality of clustering (*SI Appendix*, Fig. S26).

To investigate the spatial organization of community types, we mapped the community type labels (A through F) to the location of the corresponding crypts on the cecal tissue samples (Fig. 7*D* and *SI Appendix*, Figs. S16–S19). Interestingly, we observed that crypt communities of the same type seemed to colonize neighboring crypts. To further investigate this observation, we calculated the distance between each colonized crypt and its nearest neighbors of each type, and then we sorted the distances according to the community types involved in each pair. Crypts of type A were not considered in this analysis because they were not enriched with any particular taxonomic group. For community types B through E, we found that the median nearest-neighbor distance between crypts occupied by communities of the same type (i.e., B–B, C–C, D–D, and E–E) was smaller than when two communities of different types were involved (*SI Appendix*, Fig. S23 and Tables S5 and S6). The exception were communities of type F, because the median distance C–F (192.9 µm) was smaller than the median distance F–F (812.7 µm). We also observed that the length scale for the clustering of communities of the same type was ∼1 to 2.5 times the median distance between adjacent crypts (*SI Appendix*, Table S6). We also performed the spatial analysis of community types for unexposed and recovery mice separately (*SI Appendix*, Figs. S27 and S28 and Tables S7 and S8).

Noticeably, in one cage in which Bacteroidetes could not be detected in feces by sequencing after recovery (*SI Appendix*, Table S3), 3D imaging showed that 5% of crypts (3 out of 56) were of type D, which was defined by a high abundance of Clostridia and Bacteroidetes. To resolve the discrepancy between the sequencing of fecal microbiota and the 3D imaging of mucosal bacteria about the presence of Bacteroidetes in this cage after recovery, we carefully reexamined the corresponding images and their analysis. We found that the probes for Clostridiaceae and Bacteroidetes overlapped in some cells, suggesting the cfb560 probe hybridized off its intended target in that cage (*SI Appendix*, Fig. S20). Approximately half of the crypts in that sample had objects in the cfb560–A546 channel for Bacteroidetes. However, they were relatively small, with median and average volumes of 0 and 78 voxels respectively, whereas, in the other two recovery cages, where we did not find evidence of off-target hybridization (*SI Appendix*, Fig. S21), the median and average were 101 and 252.2 voxels. Moreover, in mice unexposed to ciprofloxacin, the median and average volumes of objects classified as Bacteroidetes were 276 and 585.3 voxels, respectively. Therefore, despite off-target hybridization of probe cfb560, 3D imaging confirmed that Bacteroidetes were least abundant in the cage where they were undetected by sequencing.

An early version of this work was included and cited in the thesis of coauthor Roberta Poceviciute (51).

## Discussion

This article presents a technical approach to investigate the biogeography of the native intestinal microbiota in fixed tissue. By systematically reconciling 3D imaging and tissue clearing with multiplexed staining of bacterial rRNA, we enabled the measurement of the composition of the mucosal microbiota with taxonomic and high spatial resolution. The large size of samples in whole-mount display enabled mapping of bacteria over the scale of centimeters to microns. This is a valuable capability because the physical and biological interactions that may shape the spatial structure of microbial communities take place over a wide range of spatial scales. In the context of the recovery of the microbiota after antibiotics, we used 3D imaging with taxonomic resolution to investigate how disrupting the microbiota with a wide-spectrum antibiotic might modify the patterns of bacterial colonization of cecal crypts.

The thin hydrogel film used to preserve large areas of exposed mucosa was effective at protecting tissue-associated bacteria in all segments of the gut. This protective film enabled processing the delicate gut tissues for 3D imaging of native bacteria, mucus, and host cells. In the cecum, we found widespread colonization of crevices (created by merging crypts) and patchy colonization of crypts. Similarly, a large portion of the proximal colon of mice was uniformly covered by layers of mucus and bacteria that are analogous to the dual mucus layer previously observed through 2D imaging in the colon (36, 38). Given the ability of our method to repeatedly recover the layered mucus interface of the proximal colon, we believe that our observations in the cecum accurately reflect the colonization of cecal crypts by bacteria. However, orthogonal imaging methods that use other preservation agents and strategies, such as scanning electron microscopy, could be used to further evaluate the physiological relevance of our observations. Another factor that may limit the repeatability of our observations is the variability across individual mice. Indeed, because we did not image mice of cohort A at day 0, we imaged mice that were not exposed to ciprofloxacin in cohort B, assuming that both groups would display equivalent mucosal colonization because they had the same age and origin, and a similar microbiota. However, this assumption would require further testing to be validated.

The perception of spatial phenomena depends strongly on the scale and mode of observation. Prior studies have used imaging to show that intestinal microbes can colonize crypts (2, 30, 52). However, these studies provided a limited view of mucosal bacteria, or the extent of the surveyed tissue was not clear. Therefore, our observation that cecal crypts were colonized by a complex microbiota in patches that spanned several length scales was surprising, and highlighted the importance of imaging large areas of tissue in 3D to measure the extent of mucosal colonization. The ability to measure crypt colonization may provide further insights into the ability of specific microbes to colonize the intestinal interface and into the ability of the host to regulate colonization. For instance, based on *A. muciniphila*’s ability to utilize mucus in vitro, we expected that it would stand out as a colonizer of crypts. Instead, we made the counterintuitive observation that only one crypt out of hundreds of crypts with microbes was clearly colonized by *A. muciniphila*. Additionally, the ability to measure the highly regulated mucosal colonization may be of use to study host–microbe interactions. For instance, despite the high concentration of bacteria in the cecum of mice and the absence of a thick layer of mucus to segregate bacteria from the cecal epithelium, we were intrigued by the observation that the majority of crypts were not colonized by bacteria, potentially due to barriers set up by the host.

Our 3D imaging method provided the means to examine the taxonomic makeup of individual crypts and allowed us to see closer into the spatial organization of crypt communities. Preliminary 3D imaging and sequencing showed that bacteria of the family Muribaculaceae might play an important role in the patchy colonization of crypts (Figs. 4 and 5). Also, finding Firmicutes near the luminal end of crypts suggested that a combination of host or ecological factors might limit their depth of crypt penetration. To test whether the elimination of Muribaculaceae would change these spatial patterns, we exposed the microbiota of mice to the wide-spectrum antibiotic ciprofloxacin. The antibiotic cleared crypts from bacteria after 4 d, and Muribaculaceae were undetectable after 10 d without antibiotic. In the absence of Muribaculaceae and antibiotic, cecal crypts became recolonized in a manner comparable to the colonization of crypts that were unexposed to antibiotics. We also found that Bacilli (Lactobacillaceae) were closer to the luminal end of crypts than Bacteroidetes and Clostridia, regardless of the exposure to ciprofloxacin.

One key feature of the workflow presented here is the supplementation of FISH with multiplexed orthogonal signal amplification (HCR) (44) and spectral imaging (53). HCR provided 3D imaging with a high signal/background ratio, and allowed us to use off-the-shelf FISH probes despite their cross-reactivity (Fig. 5*B* and *SI Appendix*, Fig. S20). The specificity of probes and the multiplexing capacity of the method will be further increased by the use of third-generation HCR probes and even more orthogonal HCR initiator sequences (54). Also, the set of FISH probes that we used is not intrinsic to the method presented here, and taxon-specific probes could be designed de novo like in recent 2D imaging of fecal microbiota (19, 20). Another key feature of the method is the extensive imaging of large samples, which enabled the acquisition of enough data to reveal spatial patterns. However, imaging of large samples and the analysis of 3D images is slow and has many manual components; therefore, future efforts should be directed toward automated image processing and analysis of 3D imaging.

The ability to measure the taxonomic makeup of hundreds of crypt communities at the tissue scale allowed us to examine the spatial distribution of bacterial communities according to their taxonomic makeup. By measuring the distance between communities of different taxonomic types, as defined by a hierarchical clustering algorithm, we found that communities with similar taxonomic composition were closer to each other than dissimilar ones. Similar separate analyses for samples at different stages of antibiotic exposure showed that spatial correlations in the taxonomic composition of crypt communities are more clearly defined (statistically significant) in naïve samples than in samples that recovered from antibiotics. Additional insights about the spatial correlation of the taxonomic composition of crypt communities may be derived by analyzing multivariate Mantel correlograms, as thoughtfully suggested by a reviewer. Future work will help disentangle how the propagation of bacterial colonization from crypt to crypt and host-driven heterogeneities drive spatial taxonomic correlations.

In summary, taxonomically resolved 3D imaging of mucosal microbes enabled the identification of patterns in their spatial distribution at different scales and allowed us to recognize spatial patterns that did not change after the elimination of a dominant bacterium and the recolonization of crypts. Therefore, we propose that this method, in tandem with fine manipulations of the composition of intestinal microbes and host genetics, provides a platform to study host–microbe interactions and bacterial ecology. Biogeographical analysis of the mucosal microbiota of the gut is of special interest in the context of intestinal diseases in which the microbiota is causally involved but specific microbes and mechanisms by which they act have not been implicated. The methods presented here can be expanded to understand how other elements of the microbiota, like microscopic fungi, protozoa, and intestinal parasites, interact with bacteria and the host to coexist.

## Materials and Methods

### Sources and Strains of Laboratory Mice.

Intestinal tissue for sequencing and imaging was obtained from adult male SPF C57BL/6J mice (The Jackson Laboratory). In the rearing facility at California Institute of Technology (Caltech), SPF mice were housed four to a cage and given sterile food and water ad libitum. SPF mice were sourced from the same room at the provider’s facility to minimize the environmental sources of variability in the microbiota of mice. GF tissue for imaging was obtained from a male mouse from a colony of gnotobiotic mice with B6 background maintained at Caltech. All animal work was performed in accordance with Caltech Institutional Animal Care and Use Committee protocols #1646 and #1769.

### Tissue Preservation and Clearing for Imaging.

Tissues for imaging of the mucosal microbiota were prepared as detailed in *SI Appendix*, *Supplementary Materials and Methods*. Briefly, samples went through the following treatments sequentially. 1) C57BL/6J mice of 20 wk to 21 wk old or 13 wk to 15 wk old were euthanized by transcardial perfusion of cold saline. 2) Intestines were cut open longitudinally, and the bulk contents were cleared with sterile tweezers and the gentle application of sterile phosphate-buffered saline (PBS). 3) Clean tissues in whole mount were fixed in 4% paraformaldehyde for ∼1 h. 4) In an anaerobic chamber, tissues were floated for 15 min on a pool of monomer mix with the muscle side facing up, so that components of the mix could penetrate the bacterial biofilms and other contents on the tissues. The monomer mix was removed using a pipette, and the sample was incubated at 37 °C in an anaerobic chamber for 3 h to form the acrylamide gel layer at the glass–tissue interface. 5) The muscle side of tissue samples was embedded in an acrylamide matrix without bisacrylamide. This step is necessary to turn the tissue matrix into a hydrogel. Embedding lasted 3 h, after which the excess acrylamide mix was removed, and the tissue was polymerized for 3 h at 37 °C. 6) Tissue samples were removed from the glass slides with a sterile razorblade and glued onto a piece of semirigid plastic. 7) Before clearing, bacteria in gels were permeabilized according to the parameters prescribed by the optimization of lysozyme treatment (*SI Appendix*, *Supplementary Materials and Methods*). 8) Permeabilized samples were enclosed in tissue cassettes and cleared for 4 d in 8% wt/vol sodium dodecyl sulfate (SDS) in PBS, pH = 8.3 at 37 °C. SDS was vigorously stirred. SDS was removed by washing in stirred 1× PBS for 2 d at 25 °C.

### HCR Staining of Bacterial 16S rRNA.

We designed HCR probes (*SI Appendix*, Tables S1 and S2) and used them to image the location of total bacteria and specific taxa on intestinal tissue. The specific reagents and treatments used for HCR staining are described in detail in *SI Appendix*, *Supplementary Materials and Methods*.

### Imaging of Tissues.

#### Microscopy.

In situ imaging of the mucosal microbiota was carried out with an upright Zeiss LSM 880 laser-scanning confocal microscope capable of spectral acquisition and of housing a CLARITY optimized long-working-distance objective. The objectives and lasers used for the acquisition at different scales are specified in *SI Appendix*, *Supplementary Materials and Methods*.

#### Mounting medium.

For imaging with a 5× objective, samples were mounted either in 1× PBS or in RIMS and protected with a coverslip to prevent evaporation. For imaging with the refractive index–matched 20× objective, samples were always mounted in RIMS. Samples were saturated in RIMS for at least 10 h with gentle shaking before imaging. We added a layer of immersion oil on top of the pool of RIMS to prevent water evaporation and maintain a constant refractive index during experiments (16916-04, Electron Microscopy Sciences). RIMS was prepared following an available protocol (40). We substituted Histodenz by Iohexol (CAS 66108-95-0, Janestic Co., Ltd.). RIMS had a refraction index *n* ≈ 1.46 according to measurements with a digital refractometer (#13950000, AR2000, Reichert Analytical Instruments).

### Image Processing and Analysis.

#### Computational image processing.

Image stacks of mucosal bacteria obtained by in situ confocal imaging were visualized and processed in commercial software (Vision4D 3.0, Arivis AG). A detailed account of computational image processing is provided in *SI Appendix*, *Supplementary Materials and Methods*.

#### Statistical analysis of bacterial abundance and spatial distribution.

Abundance of bacteria in crypts was determined as the voxel count of probes targeting each taxon, and the abundance of each taxon across crypts was normalized by Z scoring the voxel count for each channel. Z scoring allowed the counts between channels to be more representative for the actual bacterial abundances. Individual crypts were treated as a volumetric unit hosting the bacteria, and HCA was performed to study the relationship (coexistence) of the species where both the crypts and bacterial taxa were clustered based on their cosine similarities. The six most prominent branches of the clustered groups were chosen for further analysis and mapped back into the spatial context showing the distributions of these community types in the cecum. Dimension reduction methods t-SNE and UMAP (55, 56) were used for dimension reduction to show the relationship between the branches and the cosine pairwise distance metrics.

To find the clusters of colonized crypts in a sample, we first mapped the (*x*, *y*) coordinates of the segmented crypts to the reference framework of the sample. Then, we applied a publicly available MATLAB function to the ensemble of (*x*, *y*) coordinates of crypts in each sample (57); the clustering in this function is based on the distance between points. Each point was clustered with the nearest neighbor if they were less than 150 μm apart.

The statistical Wilcoxon rank sum test and paired *t* test were evaluated with the instructions “ranksum()” and “ttest()” in Matlab.

Computations were performed with a DELL XPS 9560 with Intel(R) Core(TM) i7-7700HQ CPU and 32.0 GB of RAM on Microsoft Windows 10 Enterprise operating system. MATLAB v. R2019a was used for the data analyses.

### DNA Extraction, Sequencing, Bioinformatics Analyses, and Absolute Quantification.

DNA was extracted from murine feces and intestinal contents in house or at Zymo Research. DNA extracts were further processed for sequencing and absolute quantification, and analyzed with the ZymoBIOMICS Targeted Sequencing Service (Zymo Research). These procedures are described in *SI Appendix*, *Supplementary Materials and Methods*.

## Supplementary Material

Supplementary File

Supplementary File

Supplementary File

Supplementary File

Supplementary File

## Data Availability

Raw data have been deposited in CaltechDATA (https://data.caltech.edu/records/20077) (58).

## Change History

May 9, 2022: The data availability statement has been updated.
